# Advancing research integrity: a programme to embed good practice in Africa

**DOI:** 10.11604/pamj.2019.33.298.17008

**Published:** 2019-08-13

**Authors:** Anke Rohwer, Elizabeth Wager, Taryn Young

**Affiliations:** 1 Centre for Evidence-based Health Care, Division of Epidemiology and Biostatistics, Faculty of Medicine and Health Sciences, Stellenbosch University, Cape Town, South Africa; 2 Sideview, Princes Risborough, UK; 3 School of Medicine, University of Split, Split, Croatia

**Keywords:** Research integrity, Africa, institution, publication policy, workshop

## Abstract

In Africa, training programmes as well as institutional policies on research integrity are lacking. Institutions have a responsibility to oversee research integrity through various efforts, including policies and training. We developed, implemented and evaluated an institutional approach to promote research integrity at African institutions, comprising a workshop for researchers ("bottom-up") and discussions with senior faculty on institutional policies ("top-down"). During the first day, we facilitated a workshop to introduce research integrity and promote best practices with regards to authorship, plagiarism, redundant publication and conflicts of interest. We used a variety of interactive teaching approaches to facilitate learning, including individual and group activities, small group discussions and case-based learning. We met with senior faculty on the following day to provide feedback and insights from the workshop, review current institutional policies and provide examples of what other research groups are doing. We evaluated the process. Participants actively engaged in discussions, recognised the importance of the topic and acknowledged that poor practices occurred at their institution. Discussions with senior researchers resulted in the establishment of a working group tasked with developing a publication policy for the institution. Our approach kick-started conversations on research integrity at institutions. There is a need for continued discussions, integrated training programmes and implementation of institutional policies and guidelines to promote good practices.

## Introduction

In Africa, efforts to promote research integrity are limited, and policies as well as training initiatives at academic institutions are lacking [1-4]. While there are some training programmes on research ethics, these generally focus on ethics related to human and animal participants of studies and do not include topics linked to research integrity, responsible conduct of research (RCR) or research reporting, and formal training on RCR is lacking [2,5]. Academic institutions have a responsibility to oversee research integrity, especially in countries where national regulatory bodies and policies are lacking [1,3,6]. Efforts to promote research integrity should be multi-faceted and should include clear policies that outline best practices, handling of allegations of research misconduct, as well as consequences of research misconduct; continued awareness raising and training of all students and researchers [6]. As part of a bigger project to gain more understanding on research integrity in low- and middle-income countries (LMICs), we recently conducted a survey amongst Cochrane authors based in LMICs to analyse the perceived prevalence of poor reporting practices related to authorship, redundant publication, plagiarism and conflicts of interest [7]. Survey participants reported that poor practices were common at their institutions in terms of guest authorship (77% of 198 respondents), ghost authorship (41%), text-recycling (60%), plagiarism of translated text (37%), plagiarism of ideas (43%) and not declaring financial conflicts of interest (40%). In subsequent interviews with selected participants, interviewees reported that lack of training and mentorship contributed to poor practices. In addition, very few participants were aware of the existence of institutional policies and guidelines. We developed an approach that included both training of researchers ("bottom-up") and high-level engagement in institutional policies ("top down"), aiming to embed good practices in institutions. This report describes our approach to develop, implement and evaluate an institutional approach to promote research integrity at African institutions. We focused on practices related to reporting of research, including authorship practices, plagiarism, redundant publication and conflicts of interest.

## Workshop report

### What is research integrity?

Research integrity can be defined as “honesty in reporting and communicating, reliability in performing research, objectivity, impartiality and independence, openness and accessibility, duty of care, fairness in providing references and giving credits, and responsibility for future science generations” [8]. These values and principles are fundamental to any discipline, in any setting. Research misconduct is often defined as data fabrication, data falsification and plagiarism. However, it includes a much wider spectrum of poor practices (Table 1) such as guest authorship (adding authors that have not contributed substantially to the work) and ghost authorship (omitting authors that have contributed substantially to the work) or not declaring conflicts of interest that are just as important, more relevant on a day-to-day basis and more common compared to data fabrication and falsification [9-11].

**Table 1:** Research misconduct related to reporting research

Term	Definition
Data fabrication	Making up of data and presenting it as research findings
Data falsification	Manipulating, omitting or changing research results in order to make the data look better
Plagiarism	Copying text or part of a text, an idea or an image from another source, without properly referencing the source and using it as one’s own.
Redundant publication	Republishing one’s own work including copying of an entire manuscript (duplicate publication), publication of parts of the results in separate papers (salami publication) and re-using of text in several publications (text-recycling).
Guest authorship	Adding authors to a manuscript who did not contribute substantially to the work.
Ghost authorship	Omitting authors who have contributed substantially to the work.
Conflicts of interest	A financial or non-financial (personal, political, academic, religious, institutional) interest that can potentially influence professional judgement and bias results.

### What was our approach?

We consulted with senior faculty at two tertiary institutions in Africa to outline the package being offered and to explore opportunities to visit these institutions. Our package included a training workshop to introduce best practices to researchers ("bottom-up") on day 1 and discussions with senior faculty on institutional policies ("top down") on day 2. We developed the workshop “*Doing the right thing: A workshop on research integrity and publication ethics*” to introduce research integrity and promote best practices in authorship, plagiarism, redundant publication and conflicts of interest (Table 2). It was accredited by the Liverpool School of Tropical Medicine (LSTM) in July 2017. We facilitated the workshop at one African academic institution in May 2017 and at another in July 2017. A researcher from the Centre of Evidence-based Health Care at Stellenbosch University (AR), as part of her PhD on research integrity in LMICs and a publication specialist from the UK (EW) who has vast experience in facilitating training on research integrity, facilitated the workshops. Participants completed a pre-workshop survey on perceptions and behaviour related to research reporting practices. The questionnaire, previously developed for the survey of Cochrane authors from LMICs [7], contained scenarios related to authorship practices, plagiarism, redundant publication and conflicts of interest (Table 3). We asked participants to indicate whether practices portrayed in the scenarios were acceptable or not, whether they themselves or someone they knew had engaged in this behaviour in the past, and whether it occurred at their institution. We used a variety of instructional methods to facilitate learning. Our approach encouraged active engagement of participants and included individual and group activities, as well as case-based instruction, all of which have been found to be effective in training of principles of research integrity [12,13]. Each participant also received a list with important websites and guidelines related to publication ethics and research integrity. We asked participants to complete an evaluation form at the end of the workshop. On the day following the workshop, we had a discussion with senior faculty members, including the deans of relevant faculties, the provost and a few other senior researchers, all of whom attended the research integrity workshop. The aim of the meeting was to provide feedback and insights from the workshop, review current institutional policies and guidelines and provide examples of what other research groups and institutions are doing.

**Table 2:** summary of research integrity workshop

**Name of workshop**	Doing the right thing: A workshop on research integrity and publication ethics
**Aim**	To introduce research integrity and its importance in health research and to promote best practice in authorship attribution, conflicts of interest and avoiding plagiarism.
**Learning objectives**	After the workshop, participants will be able to: Discuss research integrity and how it relates to reporting their research Find and apply current guidelines for good research reporting practice related to authorship, conflicts of interest and plagiarism
**Participants**	Junior and senior health researchers, who want to publish in national and international journals including Masters and PhD students as well as postdoctoral researchers
**Setting**	Institutions in Sub-Saharan Africa
**Duration**	4 hours
**Teaching approach**	Interactive workshop Using scenarios on research reporting practices as a springboard for discussions Small group discussions
**Programme**	*Pre-workshop* Complete online questionnaire *Workshop* Why research integrity isn’t just somebody else’s problem Authorship, based on questionnaire scenarios Conflicts of interest, based on the questionnaire scenarios Plagiarism, based on the questionnaire scenarios Redundant publication, based on the questionnaire scenarios How to promote integrity at individual level and group level

**Table 3:** scenarios used to facilitate learning

Research reporting practice	Scenario
Guest authorship	A junior researcher, J, adds the head of department, D, as the last author on a research paper. D provided suggestions for direction of J’s work that helped her obtain the grant, although he hasn’t contributed to the actual research or the publication.
	A professor, M, who did not contribute to study design, data collection or data analysis but is an expert in the field, reviews the draft manuscript and suggests some minor changes to the English. He asks to be listed as an author on the paper.
Ghost authorship	A researcher, S, contributes to the design and does most of the data collection in a study but goes on maternity leave as it is being analysed. When she returns to her post she discovers that the research has been published by her supervisor without her name or any acknowledgement of her contributions.
Acknowledgement practices	A Master’s student consults with the resident biostatistician, P, to help with data analysis on her research project. In the manuscript that she submits for publication, she lists P in the “Acknowledgement” section.
Text-recycling/redundant publication	A PhD student “copies and pastes” nearly all of the introduction from a paper that she has previously published into her next manuscript, since she is doing a series of experiments on the same topic.
Plagiarism	A researcher in Mozambique wants to submit his manuscript to a journal published in English. He finds a text book in Portuguese that explains an aspect of the background to the disease very well. He translates one paragraph into English, and puts this into his introduction without reference to the book.
	A researcher from India attends an international conference where a European research study with a novel design is presented. He submits a protocol for an identical study to the ethics committee at his home institution. He does not reference the European study.
Conflicts of interest	A researcher, T, is working on a diagnostic test study. The company manufacturing the test has supplied the kits for free but did not design or fund the research. T was paid for a consultancy for the same company two years ago. In the publication of the study, he declares that he has no conflicts of interest.
	A researcher, K, writes a review for treatment guidelines of herbal remedies for children’s cough. K’s wife is employed by the company that manufactures one of these remedies. In the review, K declares that he has no conflicts of interest.

### What was our experience?

Workshop participants comprised mostly junior researchers in one institution and mostly senior researchers in another. However, in both workshops, participants recognised poor practices at their institution and they equally appeared enlightened when we shared guidelines on authorship and explained conflicts of interest and redundant publication in more detail. Participants actively engaged in small group discussions, which allowed them to share personal experiences and discuss given scenarios in more detail. Indeed, using scenarios to kick-start discussions worked very well in both institutions, enabling participants to have a common understanding of the issues, which most of them could relate to. Participants also commented on the usefulness of the scenarios, and suggested that further examples should be added. Although interaction was good throughout the workshop, participants were particularly vocal about authorship issues and the scenarios on guest authorship provoked lively discussions. This was not unexpected, as Cochrane authors also spoke extensively about authorship problems they encountered in their institutions [7]. Most participants were unaware of existing guidelines and found the International Committee of Medical Journal Editors criteria for authorship very useful [14]. The meeting with senior researchers was beneficial in clarifying the value of institutional policies and outlining the content of such policies. We used the publication policy of the LSTM as an example and introduced and perused the *International Standards for authors* [15], that addresses some additional issues and can be used to inform an institutional policy. One of the institutions was in the process of drafting a university-wide research policy that covered various aspects of conducting research, at the time the workshop was being held. Attendees of the meeting felt that this would be an ideal opportunity to include aspects related to research integrity and discussed the possibility of developing a policy at departmental or faculty level as a starting point. As this would be easier to implement and monitor, it could act as a pilot for an institution-wide policy. Participants agreed to form a working group that would provide input into the proposed policy.

## Conclusion

Our combined “bottom-up” and “top-down” approach worked well to initiate conversations on research integrity at institutions. Participants recognised the importance of continuing discussions as well as training in this regard. Our workshop aimed to introduce research integrity and certain reporting practices. Although participants found the workshop very useful, there is much more to be done. In addition to having more awareness-raising workshops like ours, education on the responsible conduct of research should be embedded in under- and post-graduate health programmes. Once-off training is not nearly enough to change existing cultures at institutions. Indeed, it should become an integral part of health researchers' training programmes rather than an add-on. Integrating such training in existing programmes affords the opportunity for best practices to become the “norm and to promote cultural change in research” [16]. Buy-in from senior faculty and institutions was vital in operationalising our approach. This is difficult to plan and influence, and making use of existing collaborations proved vital. As research misconduct is a sensitive topic and research integrity is poorly understood, one needs to emphasise that the aim of our programme is not to point fingers and criticise, but to improve knowledge of best practices and promote responsible conduct of research. Furthermore, buy-in from senior academics and professors, in their capacity as mentors, supervisors and role-models of students and junior researchers, adds legitimacy to training initiatives [16]. Although development of institutional policies is a vital first step, they need to be actively promoted and implemented, and discussions on research integrity should be ongoing. We are currently following-up with institutions on the development and implementation of policies.

## Competing interests

The authors declare no competing interest.

## References

[1] Kombe F, Anunobi EN, Tshifugula NP, Wassenaar D, Njadingwe D, Mwalukore S (2014). Promoting Research Integrity in Africa: An African Voice of Concern on Research Misconduct and the Way Forward. Developing world bioethics.

[2] Horn L (2016). Promoting Responsible Research Conduct: a South African Perspective. Journal of Academic Ethics.

[3] Resnik DB, Rasmussen LM, Kissling GE (2015). An international study of research misconduct policies. Account Res.

[4] Ana J, Koehlmoos T, Smith R, Yan LL (2013). Research misconduct in low-and middle-income countries. PLoS medicine.

[5] Rossouw TM, Van Zyl C AP (2014). Responsible conduct of research: Global trends, local opportunities. S Afr J Sci.

[6] Resnik DB, Master Z (2013). Policies and initiatives aimed at addressing research misconduct in high-income countries. PLoS medicine.

[7] Rohwer A, Young T, Wager E, Garner P (2017). Authorship, plagiarism and conflict of interest: views and practices from low/middle-income country health researchers. BMJ open.

[8] (ALLEA) AEA (2017). European Code of Conduct for Research Integrity - Revised Edition.

[9] Vries DER, Anderson MS, Martinson BC (2006). Normal Misbehaviour: Scientists Talk about the Ethics of Research. J Empir Res Hum Res Ethics.

[10] Martinson B, Anderson M, de Vries R (2005). Scientists behaving badly. Nature.

[11] Bouter LM, Tijdink J, Axelsen N, Martinson BC, ter Riet G (2016). Ranking major and minor research misbehaviors: results from a survey among participants of four World Conferences on Research Integrity. Research Integrity and Peer Review.

[12] Todd EM, Torrence BS, Watts LL, Mulhearn TJ, Connelly S, Mumford MD (2017). Effective Practices in the Delivery of Research Ethics Education: A Qualitative Review of Instructional Methods. Account Res.

[13] Watts LL, Medeiros KE, Mulhearn TJ, Steele LM, Connelly S, Mumford MD (2016). Are Ethics Training Programs Improving? A Meta-Analytic Review of Past and Present Ethics Instruction in the Sciences. Ethics & Behavior.

[14] ICMJE (2016). Recommendations for the conduct, reporting, editing and publication of scholarly work in medical journals.

[15] Wager E, Kleinert S, Mayer T, Steneck N (2011). Responsible research publication: international standards for authors, A position statement developed at the 2nd World Conference on Research Integrity, Singapore, July 22-24, 2010.

[16] Hooper M, Barbour V, Walsh A, Bradbury S, Jacobs J (2018). Designing integrated research integrity training: authorship, publication, and peer review. Research Integrity and Peer Review.

